# Loneliness in Older Adults Receiving Hemodialysis and Associated Factors: A Cross-Sectional Study

**DOI:** 10.1097/jnr.0000000000000702

**Published:** 2025-10-28

**Authors:** Yeu-Hui CHUANG, Shu-Ching LU, Chih-Yu WANG, Hui-Chuan HUANG, Sophia H. HU, Shou-Yu WANG

**Affiliations:** 1 School of Nursing, College of Nursing, Taipei Medical University, Taipei, Taiwan; 2 Department of Nursing, Wan Fang Hospital, Taipei Medical University, Taipei, Taiwan; 3 Research Center in Nursing Clinical Practice, Wan Fang Hospital, Taipei Medical University, Taipei, Taiwan; 4 Cardinal Tien Hospital, New Taipei City, Taiwan; 5 College of Nursing, The Ohio State University, Columbus, OH, USA; 6 Department of Nursing, College of Nursing, National Yang Ming Chiao Tung University, Taipei, Taiwan; 7 School of Nursing and Midwifery, College of Health, Medicine and Wellbeing, University of Newcastle, Callaghan, NSW, Australia; 8 School of Health, Faculty of Medicine and Health, University of New England, Armidale, NSW, Australia; 9 School of Nursing and Midwifery, University of Southern Queensland, Ipswich, QLD, Australia; † Contributed equally

**Keywords:** loneliness, older patients, hemodialysis, well-being

## Abstract

**Background:**

The number of patients with end-stage renal disease (ESRD) in Taiwan continues to rise steadily, and hemodialysis is the primary treatment for these patients. More than 50% of patients on hemodialysis are over 65 years of age, indicative of an aging patient population. Changes in lifestyle, dietary habits, and social roles after commencing hemodialysis easily cause patients to feel uncertain, depressed, and/or lonely. However, a limited number of studies have examined the issue of loneliness among older adults on hemodialysis.

**Purpose:**

This study was designed to better understand loneliness and its associated factors among older patients receiving hemodialysis.

**Methods:**

A cross-sectional research design with convenience sampling was employed with a total of 146 patients. Eligible participants aged 65 years or older and receiving hemodialysis for more than 3 months were recruited from two hospitals in northern Taiwan. Structured questionnaires, including a demographic characteristics questionnaire, the Hemodialysis Patient Fatigue Scale, Social Support Scale, and UCLA Loneliness Scale version 3, were used to collect data.

**Results:**

The mean loneliness score was 41.5 out of a maximum of 80. A significant positive relationship was found between fatigue and loneliness, and a significant negative relationship was found between social support and loneliness. Gender, fatigue, and social support were identified as predictors of loneliness.

**Conclusions/Implications for Practice:**

The results indicate that older patients receiving hemodialysis have a moderate level of loneliness. Based on the identified predictors, nurses should pay particular attention to patients who are female, are experiencing a higher fatigue level, and/or have lower social support. Future studies may use these results to develop strategies for preventing and improving loneliness among older adults receiving hemodialysis.

## Introduction

End-stage renal disease (ESRD) is a common and serious disease affecting public health in Taiwan. United States Renal Data System (2025) data show that Taiwanese had the highest incidence and prevalence of treated ESRD in the world in 2022. Common treatments for ESRD include hemodialysis, peritoneal dialysis, and renal transplantation. Before renal transplantation, most patients in Taiwan receive dialysis to sustain their lives, with 92.5% receiving hemodialysis and 7.5% receiving peritoneal dialysis (Taiwan Society of Nephrology, 2024). However, renal transplantation in Taiwan is still a challenge. Kidney transplant waiting rates were 8.5%–9.1% in 2012–2021, but the rate of transplantation in 2021 (the number of patients receiving transplantation/the number of patients waiting for transplantation) was only 3.9% (Taiwan Society of Nephrology, 2024). Therefore, in Taiwan, hemodialysis is still the prevalent choice for patients with ESRD, especially those aged 65 years or over. According to the 2023 Kidney Disease Annual Report in Taiwan, the crude prevalence rate of dialysis in 2021 was 3,839 per million population (Taiwan Society of Nephrology, 2024). Moreover, the number of patients aged over 65 years on dialysis increased from 39,978 in 2016 to 50,436 in 2021. These statistics also show that the mean age of patients receiving hemodialysis for the first time was 68.5 years in 2021. In light of these data, the number of older patients on hemodialysis is growing year by year in Taiwan.

While hemodialysis helps sustain life, these older patients must also deal with the negative effects associated with this disease and its treatment such as fatigue, stress (Kang & Chae, 2021), itchy skin (Daraghmeh et al., 2022), depression (Liu et al., 2024; Othayq & Aqeeli, 2020), frailty (Imamura et al., 2023), increased hospitalization rates, and higher risk of mortality (Hall et al., 2018). Previous findings indicate that older patients receiving dialysis experience physical and psychological discomfort associated with the disease and treatment, and experience changes in lifestyle habits, dietary patterns, and social roles in the long-term therapy process. Furthermore, patient uncertainty about their illness may reduce their self-efficacy and self-care compliance, influencing their emotional and mental health (Kim & Kim, 2019; Nguyen et al., 2022).

Loneliness is a subjective negative experience that includes feelings of frustration, helplessness, abandonment, and being unwelcome; difficulties with interpersonal and social interactions; a sense of emptiness and negativity; and a lack of meaning and value in life (Russell, 1996). Loneliness is an increasingly common problem in the world that can lead to worsening conditions in patients with chronic diseases and may also indirectly influence their physical health (Kanbay et al., 2023), psychological, and social health (Mann et al., 2022). Although prior studies have reported that patients receiving hemodialysis experience moderate levels of loneliness, these studies did not specifically focus on older patients. For example, Akin et al. (2014) included patients with a mean age of 55.88±15.56 (range: 15–91) years, while Pallone et al. (2022) recruited patients with a mean age of 59.63±15.14 (range: 27–85) years. A possible reason may be that the patients in these studies were less likely to participate in social activities due to exhaustion (Akin et al., 2014). In addition, the disease or their physical condition may have prompted them to withdraw, engage in self-pity, easily complain, feel less confident, be unwilling to go out, or even isolate themselves from society. In contrast, a study focused on young and middle-aged hemodialysis patients (18–65 years old) reported low levels of loneliness (Saedi et al., 2019). Of the few studies in the literature that have specifically investigated loneliness in older patients receiving hemodialysis, one qualitative study found potentially elevated risks of loneliness, despair, and fear (Sahaf et al., 2017). With the number of older patients receiving hemodialysis continuing to rise, health care professionals, in addition to providing physical care and disease treatment, should give focused attention to the psychological well-being of this patient group. To date, most of the studies on mental problems faced by hemodialysis patients in the literature have focused on stress, anxiety, and depression (Kang & Chae, 2021; Othayq & Aqeeli, 2020; Schouten et al., 2019), with only a few studies on loneliness, especially among older patients. Thus, the purpose of this study was to explore loneliness and its associated factors among older patients receiving hemodialysis, with the hypothesis that demographic characteristics would predict loneliness in this population.

## Methods

### Design, Participants, and Settings

A cross-sectional research design was employed and structured questionnaires were used to collect data. A convenience sample of patients was recruited from hemodialysis units in two hospitals in northern Taiwan. The inclusion criteria were patients who (1) were 65 years old or older; (2) had been receiving hemodialysis treatment for at least 3 months; (3) had clear consciousness; and (4) were able to speak Taiwanese or Mandarin. The exclusion criteria were patients who (1) had been diagnosed with depression or another mental disease; (2) had cancer and were receiving chemotherapy or radiation therapy; or (3) were receiving hospice care. The minimum sample size was estimated to be 146 using G-power version 3.1.9 with an α of .05, a power of 0.80, and an effect size of 0.15 (Cohen, 1988). Ultimately, 146 qualified older patients were recruited and enrolled as participants in this research.

### Instruments

The instruments employed in this study included a demographic characteristics questionnaire, the Novel Fatigue Scale for Hemodialysis Patients, the Social Support Scale for Hemodialysis Patients, and the UCLA Loneliness Scale version 3.

#### Demographic Characteristics Questionnaire

This questionnaire gathered information on participant age, gender, marital status, educational level, economic status, religious beliefs, living arrangement, leisure activities, working status, comorbidities, duration of hemodialysis treatment, and level of itchy skin (1–10). Activities of daily living (ADLs) were also measured using the Barthel Index (Mahoney & Barthel, 1965). The Barthel Index is a 10-item questionnaire with a score range of 0–100 and higher scores indicating better self-care ability.

#### Novel Fatigue Scale for Hemodialysis Patients

The Novel Fatigue Scale for Hemodialysis Patients developed by Lin et al. (2006) contains 26 items scored on a four-point Likert scale (1=*rarely or never*; 2=*sometimes*; 3=*often*; 4=*almost daily*). Possible scale score totals range from 26 to 104, with higher scores indicating higher level of fatigue. The Cronbach’s α of this scale was .91 in Lin et al. (2006) and .95 in this study.

#### Social Support Scale for Hemodialysis Patients

The Social Support Scale for Hemodialysis Patients developed by Lin et al. (1995) contains 18 items scored on a four-point Likert scale (1=*never*; 2=*rarely*; 3=*sometimes*; 4=*always*). Possible scale score totals range from 18 to 72, with higher scores indicating greater social support. The Cronbach’s α of the scale was .76 in Lin et al. (1995) and .91 in this study.

#### UCLA Loneliness Scale Version 3

Chang and Yang (1999) translated the Traditional Chinese version of the UCLA Loneliness Scale version 3 developed by Russell (1996). The 20 items on this scale are scored on a four-point Likert scale (1=*never*; 2=*rarely*; 3=*sometimes*; 4=*always*). Possible scale score totals range from 20 to 80, with higher scores indicating higher levels of loneliness. Items 1, 5, 6, 9, 10, 15, 16, 19, and 20 are positive statements and thus reverse-scored. The content validity index (CVI) and Cronbach’s α of this scale were identified as .85 and .88 in Hou and Chen (2008), and the Cronbach’s α was 0.83 in this study.

### Data Collection Procedure

In the 1-hour waiting period before patient dialysis sessions during all three shifts, one of the researchers coordinated with ward nursing staff to screen patients with regard to inclusion and exclusion criteria and introduce qualified patients to the researcher. The researcher then explained the purpose, data collection method, benefits, and risks of the study to these patients. Those patients who agreed to take part in the study completed the questionnaires after signing an informed consent form. For those who could not read, the researcher read the questions aloud and assisted them in completing the questionnaires, which required about 15–20 minutes. Although the researcher was prepared to offer immediate emotional support to the participants during the questionnaire completion process if necessary, fortunately, none expressed feeling sad or having negative feelings. Data collection was conducted between March and April 2019.

### Data Analysis

Data were analyzed using SPSS/Windows, version 19.0 (SPSS, Armonk, NY, USA). Percentage, mean (*M*), standard deviation (*SD*), Pearson product-moment correlation, *t* test, and multiple linear regression were used to identify loneliness and its associated factors among the participants.

### Ethical Considerations

This study was approved by the ethics committee of the Cardinal Tien Hospital institutional review board (IRB No. CTH-107-3-5-066). All of the participants signed an informed consent form before completing the questionnaires.

## Results

### Demographic Characteristics

An overview of participants’ demographic characteristics is given in Table 1. Of the 146 older patients receiving hemodialysis enrolled in the study, half were female (*n*=73) and half were male (*n*=73). The mean age of participants was 74.56 (*SD*=7.64, range=65–99) years; over half were married (56.2%); and a majority were literate (93.2%), had retired from employment (90.4%), had religious beliefs (77.4%), were of fair economic status (81.6%), engaged in leisure activities (87.7%), and lived with their family (90.4%). Duration of hemodialysis treatment ranged from 3 to 362 months (30 years), with a mean duration of 66.94 (*SD*=64.29) months, equivalent to 5 years and 6 months. Nearly all (139; 95.2%) of the participants had concomitant chronic diseases (in addition to kidney disease), with a mean of 1.56 (*SD*=0.69) concomitant chronic diseases. Moreover, 82.9% reported having itchy skin, with an average itchiness level of 3.72 (*SD*=2.79). The total mean ADL score was 90.13 (*SD*=19.28), with scores ranging from 0 to 100.

**Table 1:** Demographic Characteristics (N=146)

Variable	*n* (%)	Range
Age (years; *M* and *SD*)	74.56 (7.64)	65–99
Gender		
Male	73 (50.0)	
Female	73 (50.0)	
Marital status		
Single, divorced, separated, or widowed	64 (43.8)	
Married	82 (56.2)	
Educational level		
Illiterate	10 (6.8)	
Literate	136 (93.2)	
Working status		
Unemployed	14 (9.6)	
Employed before but now retired	132 (90.4)	
Religious beliefs		
No	33 (22.6)	
Yes	113 (77.4)	
Economic status		
Insufficient income	27 (18.4)	
Sufficient income	119 (81.6)	
Leisure activities		
No	18 (12.3)	
Yes	128 (87.7)	
Living arrangements		
Not living with family	14 (9.6)	
Living with family	132 (90.4)	
	*M* (*SD*)	Range
Duration of hemodialysis treatment (month)	66.94 (64.29)	3–362
Number of comorbidities (excluding kidney diseases)	1.56 (0.69)	0–3
Level of skin itchiness	3.72 (2.79)	0–10
Activities of daily living	90.13 (19.28)	0–100

### Loneliness Level and Associated Factors

The average loneliness score was 41.5 (*SD*=8.32), with scores ranging from 20 to 60. The average scores for each UCLA Loneliness Scale version 3 item are presented in Table 2. No association with level of loneliness was identified for the following factors: age (*r*=.02, *p*=.781), gender (*t*=1.68, *p*=.095), marital status (*t*=−1.07, *p*=.287), educational level (*t*=0.25, *p*=.798), economic status (*t*=0.61, *p*=.541), religious beliefs (*t*=1.11, *p*=.267), living arrangements (*t*=−1.43, *p*=.154), working status (*t*=0.98, *p*=.326), leisure activities (*t*=1.11, *p*=.269), duration of hemodialysis treatment (*r*=−.02, *p*=.780), ADLs (*r*=−.02, *p*=.861), and comorbidities (*t*=−0.22, *p*=.832).

**Table 2:** Average Item Scores for the UCLA Loneliness Scale Version 3

Item	Mean±*SD*
1. How often do you feel that you are “in tune” with the people around you? ^a^	2.00±0.86
2. How often do you feel that you lack companionship?	2.12±1.01
3. How often do you feel that there is no one you can turn to?	1.82±0.80
4. How often do you feel alone?	2.01±0.93
5. How often do you feel part of a group of friends? ^a^	2.18±0.93
6. How often do you feel that you have a lot in common with the people around you? ^a^	2.46±0.81
7. How often do you feel that you are no longer close to anyone?	1.79±0.80
8. How often do you feel that your interests and ideas are not shared by those around you?	1.98±0.84
9. How often do you feel outgoing and friendly? ^a^	2.20±0.97
10. How often do you feel close to people? ^a^	2.30±0.80
11. How often do you feel left out?	1.85±0.86
12. How often do you feel that your relationships with others are not meaningful?	1.80±0.83
13. How often do you feel that no one really knows you well?	1.88±0.79
14. How often do you feel isolated from others?	1.80±0.82
15. How often do you feel you can find companionship when you want it? ^a^	2.30±0.93
16. How often do you feel that there are people who really understand you? ^a^	2.56±0.77
17. How often do you feel shy?	1.93±0.84
18. How often do you feel that people are around you but not with you?	2.21±0.84
19. How often do you feel that there are people you can talk to? ^a^	2.21±0.77
20. How often do you feel that there are people you can turn to? ^a^	2.08±0.84

^a^ Reverse: scored items, requiring reverse scoring.

The mean total score on the Novel Fatigue Scale for Hemodialysis Patients was 57.7 (*SD*=17.7), with scores ranging from 26 to 102. A significant positive correlation was identified between fatigue and loneliness (*r*=.25, *p*=.002), indicating that the level of loneliness increases with the level of fatigue. In addition, the mean total score on the Social Support Scale for Hemodialysis Patients was 54.0 (*SD*=9.04), with scores ranging from 31 to 72. A significant negative correlation was identified between social support and loneliness (*r*=−.22, *p*=.009), indicating that having a better social support system helps reduce loneliness.

### Predictors of Loneliness

The *t* test and Pearson correlation analysis results identified only fatigue and social support as significantly associated with loneliness level. However, as gender, age, marital status, economic status, living arrangements, and ADLs have been previously identified as factors with significant effects on loneliness in the literature, these variables were included in the multiple linear regression model. Three significant predictors were found, including gender, fatigue level, and social support (*F*=9.702, *p*<.001), with an adjusted *R* ^2^ value of .153 (*R* ^2^=.170; Table 3). These results show the mean loneliness score was 2.79 points higher in female than male participants; a 1-point increase in fatigue level increased the level of loneliness by 0.17 points; and a 1-point increase in social support decreased the level of loneliness by 0.25 points.

**Table 3:** Predictors of Loneliness (*N*=146)

Variable	*B*	*SE*	Beta	*t*	*p*
Constant	49.80	4.18			
Gender (ref. female)	−2.79	1.30	−0.17	−2.14	.034^*^
Fatigue	0.17	0.04	0.35	4.38	<.001***
Social support	−0.25	0.07	−0.27	−3.46	.001**

*Note*. *F*=9.702, *p*<.001, *R* ^2^=.170, adjusted *R* ^2^=.153; *B* = unstandardized regression coefficient; *SE =* standard error of the regression coefficient. ref. = reference. Gender, age, marital status, economic status, living arrangements, activities of daily living, fatigue, and social support were entered into the regression analysis.

^*^ *p*<.05. ^*^^*^ *p*<.01. ^***^ *p*<.001.

## Discussion

The findings of this study show that the participants in this study (older patients receiving hemodialysis) had a moderate level of loneliness (*M*=41.5, *SD*=8.32) based on a categorization approach used in previous research that associates scores in the 28–43 range with moderate loneliness (Lee et al., 2019). Some previous studies investigated loneliness of patients undergoing hemodialysis using the UCLA Loneliness Scale, but the population in those studies did not mainly target older adults. The average loneliness score of 38.15±13.70 in Iranian patients (range: 18–65 years old) reported by Saedi et al. (2019) was lower than that found in this study. The average score for Turkish patients aged 15–91 years old was reported as 41.73±7.84 (Akin et al., 2014) and, in Pallone et al. (2022), over half (55%) of Brazilian patients between 27 and 85 years reported a moderate level of loneliness, while 21.3% reported a high level of loneliness. Hemodialysis routine and complications may exacerbate social isolation and psychological distress in patients, limiting their social connections and affecting their perceived loneliness (Hejazi et al., 2021). Compared with nonolder adult patients receiving hemodialysis, older patients have higher loneliness scores. As older patients with chronic renal failure are more vulnerable and must adapt to hemodialysis-related changes (Pradila et al., 2021) and cope with potential social isolation (Zhou et al., 2021), health professionals should pay greater attention to the issue of loneliness in this population.

Age, marital status, economic status, living arrangements, and ADLs were not found to predict loneliness, which contrasts with the findings of other studies on older adults receiving hemodialysis. The unique challenges faced by this population due to treatment and chronic conditions may mask the previously observed significant influences of these demographic variables. Other possible reasons for these demographic variables not being identified as predictors include most participants in this study having similar living arrangements and socioeconomic backgrounds. Over 90% of participants lived with their family, had sufficient income, and had good self-care ability. To overcome this limitation, future research should investigate these relationships on larger, more diverse samples, and/or employ longitudinal studies.

In addition, being female, being more fatigued, and having less social support were each found to significantly influence the level of loneliness. The female participants in this study reported a higher mean level of loneliness than their male counterparts. This contradicts Akin et al. (2014), which reported no significant gender effect on loneliness level in hemodialysis patients. Dahlberg et al. (2022) suggested that advancing age leads to health and functional declines and that widowhood may contribute to gender differences in loneliness. Thus, future research should focus on clarifying the influence of gender-specific factors and developing gender-sensitive interventions to alleviate loneliness in hemodialysis patients.c

In addition, fatigue severity was found to be significantly and positively associated with the level of loneliness. To the best of the author’s knowledge, the association between fatigue and loneliness in hemodialysis patients has only rarely been studied. Although Akin et al. (2014) revealed that hemodialysis patients over 60 years of age had higher levels of both loneliness and fatigue than their younger peers, they did not examine the relationships between these two variables. Similar findings have since been observed in other patient groups. For example, Deckx et al. (2015) reported that older cancer patients experiencing prolonged fatigue had an elevated risk of developing feelings of loneliness. Also, Powell et al. (2021) reported that older adults who felt lonely were twice as likely to experience fatigue as those who did not. Fatigue is a common symptom among patients receiving hemodialysis. Individuals with chronic fatigue syndrome may lack energy, resulting in social withdrawal or interpersonal alienation issues, such as reduced interactivity with family and reduced participation in social activities. Furthermore, fatigue induced by a chronic illness can amplify negative emotions such as anger, pain, and unhappiness, which may exacerbate psychosocial challenges and increase the risk of emotional loneliness (Boulazreg & Rokach, 2020). In light of the bidirectional link between fatigue and loneliness in older patients receiving hemodialysis, integrated care strategies designed to meet both physical and mental health needs should be developed in future research.

In this study, social support was identified as a predictor of loneliness in older patients receiving hemodialysis, implying that older adults with better social support feel less lonely. This result is similar to the finding of Pallone et al. (2022), which showed that higher levels of social support were associated with lower levels of loneliness in 80 Brazilian hemodialysis patients with a mean age of 59.63 years.

Moreover, previous evidence indicates that social support promotes mental health in older persons with chronic diseases, enhancing their ability to cope with loneliness (Shi et al., 2023). A review article identified family, friends, neighbors, and the government as key to creating a social environment that can help overcome loneliness in older adults (Resna et al., 2022). Family affection brings joy and conveys deep values and the importance of older individuals, while neighbors provide vital support, acting as surrogate friends/family to enhance wellness and meet daily needs. In Taiwan, while adult children and their families are the primary sources of social support for older adults (Dai et al., 2016; Liu et al., 2018), their peers in Western countries rely on a broader social network that includes family members, friends, neighbors, and the community (Alexopoulou et al., 2016; Bélanger et al., 2016; Novick et al., 2024). A systematic review noted that family support had a more significant impact on depression in older adults living in Asian communities than those living in Western countries (Tengku Mohd et al., 2019). However, another study found people in East Asia to be less willing to seek social support than Westerners due to empathic concerns (feelings of sympathy and concern for others) and relational concerns (Zheng et al., 2022). Thus, health care professionals in Taiwan are encouraged to regularly assess the social support conditions of hemodialysis patients, especially the quantity and quality of support received from family members, to provide them with proper assistance and referrals to prevent or mitigate loneliness.

In Taiwan, advancements in health care have resulted in 5-year survival rates of 55.2% for dialysis patients and 90.3% for transplant patients, surpassing the rates in many other countries. In addition, Taiwan’s National Health Insurance covers the costs of hemodialysis, ensuring medical accessibility and alleviating significant financial burdens for patients (Taiwan Society of Nephrology, 2024). As a result, hemodialysis for patients with ESRD is very common in Taiwan, with treatment and care often being task-oriented. Consequently, the emotional well-being, psychological health, and social support of patients can easily be overlooked. The findings of this study remind health care professionals of the critical need to address not only the physical but also the emotional, psychological, and social needs of hemodialysis patients.

### Limitations

First, convenience sampling was used to recruit participants from two hospitals in northern Taiwan, which may limit the generalizability of findings. Second, most of the participants lived with their families and had good self-care ability and a fair economic status. These factors may confound or influence perceived loneliness (Akin et al., 2014). Thus, participants with a greater diversity in terms of family situations, economic status, and self-care ability should be recruited in future studies. Third, future research should include longitudinal studies that track changes in loneliness over time to assess the impacts of aging and hemodialysis-related factors to provide a more comprehensive understanding of how these factors interact and influence loneliness in patients. Fourth, although the data for this study were collected in 2019, the findings of this study remain relevant to current practice. The clinical protocols for hemodialysis and the health care system’s approach in Taiwan have not significantly changed, and the challenges faced by older adults receiving hemodialysis, such as fatigue and the need for social support, remain significant aspects of chronic disease management. Lastly, while the raw score differences of 0.17 points for fatigue and −0.25 points for social support may seem small individually, they have significant cumulative clinical implications for patient care, indicating nurses should be encouraged to make greater efforts to improve fatigue levels and social support in patients receiving hemodialysis. As these scores improve, patients are likely to experience less loneliness.

### Conclusions

Older patients receiving hemodialysis have a generally moderate level of loneliness, with being female, having a higher level of fatigue, and having a lower level of social support each associated with higher levels of loneliness. In addition to addressing the physiological needs of older patients receiving hemodialysis, health care professionals should pay greater attention to patients’ subjective feelings and negative emotions, such as loneliness. To address this issue, all patients should be screened for potential and current loneliness, taking into consideration gender differences, fatigue levels, and social support, to facilitate the provision of tailored interventions designed to prevent or alleviate loneliness.

## References

[R1] Akin S. Mendi B. Ozturk B. Cinper C. Durna Z. (2014). Assessment of relationship between self-care and fatigue and loneliness in hemodialysis patients. Journal of Clinical Nursing, 23(5-6), 856–864. 10.1111/jocn.12248 23808612

[R2] Alexopoulou M. Giannakopoulou N. Komna E. Alikari V. Toulia G. Polikandrioti M. (2016). The effect of perceived social support on hemodialysis patients’ quality of life. Materia Socio-Medica, 28(5), 338–342. 10.5455/msm.2016.28.338-342 27999480 PMC5149438

[R3] Bélanger E. Ahmed T. Vafaei A. Curcio C. L. Phillips S. P. Zunzunegui M. V. (2016). Sources of social support associated with health and quality of life: A cross-sectional study among Canadian and Latin American older adults. BMJ Open, 6(6), Article e011503. 10.1136/bmjopen-2016-011503 PMC4932270 27354077

[R4] Boulazreg S. Rokach A. (2020). The lonely, isolating, and alienating implications of myalgic encephalomyelitis/chronic fatigue syndrome. Healthcare, 8(4), Article 413.https://doi.org/10.3390/healthcare8040413 33092097 10.3390/healthcare8040413 PMC7711762

[R5] Chang S.-H. Yang M.-S. (1999). The relationships between the elderly loneliness and its factors of personal attributes, perceived health status and social support. The Kaohsiung Journal of Medical Sciences, 15(6), 337–347. 10.6452/kjms.199906.0337 (Original work published in Chinese) 10441941

[R6] Cohen J. (1988). Statistical power analysis for the behavioral sciences (2nd ed.). Routledge. https://doi.org/10.4324/9780203771587

[R7] Dahlberg L. McKee K. J. Frank A. Naseer M. (2022). A systematic review of longitudinal risk factors for loneliness in older adults. Aging & Mental Health, 26(2), 225–249. 10.1080/13607863.2021.1876638 33563024

[R8] Dai Y. Zhang C. Y. Zhang B. Q. Li Z. Jiang C. Huang H. L. (2016). Social support and the self-rated health of older people: A comparative study in Tainan Taiwan and Fuzhou Fujian province. Medicine (Baltimore), 95(24), Article e3881. 10.1097/md.0000000000003881 27310979 PMC4998465

[R9] Daraghmeh M. Badran M. Janajreh A. Hassan M. Taha A. A. Koni A. A. Zyoud S. H. (2022). Prevalence of pruritus associated with hemodialysis and its association with sleep quality among hemodialysis patients: A multicenter study. BMC Nephrology, 23(1), Article No. 213. 10.1186/s12882-022-02838-z 35715758 PMC9205133

[R10] Deckx L. van den Akker M. van Driel M. Bulens P. van Abbema D. Tjan-Heijnen V. Kenis C. de Jonge E. T. Houben B. Buntinx F. (2015). Loneliness in patients with cancer: The first year after cancer diagnosis. Psycho-Oncology, 24(11), 1521–1528. 10.1002/pon.3818 25914244

[R11] Hall R. K. Luciano A. Pieper C. Colón-Emeric C. S. (2018). Association of Kidney Disease Quality of Life (KDQOL-36) with mortality and hospitalization in older adults receiving hemodialysis. BMC Nephrology, 19(1), Article No. 11. 10.1186/s12882-017-0801-5 29334904 PMC5769495

[R12] Hejazi S. S. Hosseini M. Ebadi A. Alavi Majd H. (2021). Components of quality of life in hemodialysis patients from family caregivers’ perspective: A qualitative study. BMC Nephrology, 22(1), Article No. 379. 10.1186/s12882-021-02584-8 34774021 PMC8590210

[R13] Hou H. M. Chen Y. M. (2008). Loneliness and related factors among the elderly living in long-term care facilities. Taiwan Evidence-Based Nursing Association, 4(3), 212–221. 10.6225/jebn.4.3.212 (Original work published in Chinese)

[R14] Imamura K. Yamamoto S. Suzuki Y. Yoshikoshi S. Harada M. Osada S. Kamiya K. Matsuzawa R. Matsunaga A. (2023). Prevalence, overlap, and prognostic impact of multiple frailty domains in older patients on hemodialysis. Archives of Gerontology and Geriatrics, 114, Article 105082.https://doi.org/10.1016/j.archger.2023.105082 37290228 10.1016/j.archger.2023.105082

[R15] Kanbay M. Tanriover C. Copur S. Peltek I. B. Mutlu A. Mallamaci F. Zoccali C. (2023). Social isolation and loneliness: Undervalued risk factors for disease states and mortality. European Journal of Clinical Investigation, 53(10), Article e14032. 10.1111/eci.14032 37218451

[R16] Kang H. Chae Y. (2021). Effects of integrated indirect forest experience on emotion, fatigue, stress and immune function in hemodialysis patients. International Journal of Environmental Research and Public Health, 18(4), Article 1701. 10.3390/ijerph18041701 33578807 PMC7916581

[R17] Kim B. Kim J. (2019). Influence of uncertainty, depression, and social support on self-care compliance in hemodialysis patients. Therapeutics and Clinical Risk Management, 15, 1243–1251. 10.2147/tcrm.S218934 31695397 PMC6815211

[R18] Lee E. E. Depp C. Palmer B. W. Glorioso D. Daly R. Liu J. Tu X. M. Kim H. C. Tarr P. Yamada Y. Jeste D. V. (2019). High prevalence and adverse health effects of loneliness in community-dwelling adults across the lifespan: Role of wisdom as a protective factor. International Psychogeriatrics, 31(10), 1447–1462. 10.1017/s1041610218002120 30560747 PMC6581650

[R19] Lin C.-C. Ko N.-Y. Tsai L.-C. Chen C.-H. (1995). Assessing the effect of health belief, knowledge, and social support on compliance behaviors in chronic hemodialysis patients. The Kaohsiung Journal of Medical Sciences, 11(8), 470–480. 10.6452/kjms.199508.0470 (Original work published in Chinese) 7674428

[R20] Lin C.-C. Lee Y.-H. Hung C.-C. Lee B.-O (2006). Development of a novel fatigue scale for hemodialysis patients. Formosan Journal of Medicine, 10(4), 422–428. 10.6320/fjm.2006.10(4).02 (Original work published in Chinese)

[R21] Liu Y. M. Chang H. J. Wang R. H. Yang L. K. Lu K. C. Hou Y. C. (2018). Role of resilience and social support in alleviating depression in patients receiving maintenance hemodialysis. Therapeutics and Clinical Risk Management, 14, 441–451. 10.2147/tcrm.S152273 29535526 PMC5840278

[R22] Liu H.-H. Wu C.-L. Chiang Y.-C. Tsai K.-H. Chu T.-L. Hsiao Y.-C. (2024). Religion and spiritual health in patients with and without depression receiving hemodialysis: A cross-sectional correlational study. The Journal of Nursing Research, 32(1), Article e309. 10.1097/jnr.0000000000000592 38190331

[R23] Mahoney F. I. Barthel D. W. (1965). Functional evaluation: The Barthel index: A simple index of independence useful in scoring improvement in the rehabilitation of the chronically ill. Maryland State Medical Journal, 14, 61–65. 14258950

[R24] Mann F. Wang J. Pearce E. Ma R. Schlief M. Lloyd-Evans B. Ikhtabi S. Johnson S. (2022). Loneliness and the onset of new mental health problems in the general population. Social Psychiatry and Psychiatric Epidemiology, 57(11), 2161–2178. 10.1007/s00127-022-02261-7 35583561 PMC9636084

[R25] Nguyen T. T. N. Liang S. Y. Liu C. Y. Chien C. H. (2022). Self-care self-efficacy and depression associated with quality of life among patients undergoing hemodialysis in Vietnam. PLOS One, 17(6), Article e0270100. 10.1371/journal.pone.0270100 35709232 PMC9202875

[R26] Novick T. K. Osuna M. Emery C. Barrios F. Ramirez D. Crews D. C. Jacobs E. A. (2024). Patients’ perspectives on health-related social needs and recommendations for interventions: A qualitative study. American Journal of Kidney Diseases, 83(6), 739–749. 10.1053/j.ajkd.2023.11.005 38218454 PMC11116062

[R27] Othayq A. Aqeeli A. (2020). Prevalence of depression and associated factors among hemodialyzed patients in Jazan area, Saudi Arabia: A cross-sectional study. Mental Illness, 12(1), 1–5. 10.1108/mij-02-2020-0004 32742625 PMC7370954

[R28] Pallone J. M. Santos D. G. M. D. Oliveira Dias A. L. Ferreira L. G. S. Costa da Silva C. Orlandi F. S. (2022). Loneliness level and its associated factors in patients with hemodialysis. Clinical Nursing Research, 31(6), 1164–1171. 10.1177/10547738211061447 34955033

[R29] Powell V. D. Abedini N. C. Galecki A. T. Kabeto M. Kumar N. Silveira M. J. (2021). Unwelcome companions: Loneliness associates with the cluster of pain, fatigue, and depression in older adults. Gerontology and Geriatric Medicine, 7, 1–10. 10.1177/2333721421997620 PMC7907946 33709010

[R30] Pradila D. A. Satiadarma M. P. Dharmawan U. S. (2021). The resilience of elderly patients with chronic kidney disease undergoing hemodialysis. In Proceedings of the International Conference on Economics, Business, Social, and Humanities (ICEBSH 2021; 1191–1196). Atlantis Press. 10.2991/assehr.k.210805.187

[R31] Resna R. W. Widianti W. Nofiantoro W. Iskandar R. Ashbahna D. M. Royani R. Susilawati S. (2022). Social environment support to overcome loneliness among older adults: A scoping review. Belitung Nursing Journal, 8(3), 197–203. https://doi.org/10.33546/bnj.2092 37547116 10.33546/bnj.2092 PMC10401387

[R32] Russell D. W. (1996). UCLA Loneliness Scale (Version 3): Reliability, validity, and factor structure. Journal of Personality Assessment, 66(1), 20–40. 8576833 10.1207/s15327752jpa6601_2

[R33] Saedi F. Barkhordari-Sharifabad M. Javadi-Estahbanati M. Fallahzadeh H. (2019). Sexual function, social isolation, loneliness and self-esteem in patients undergoing hemodialysis. Sexuality and Disability, 37(3), 401–413. 10.1007/s11195-019-09575-6

[R34] Sahaf R. P. Sadat Ilali E. P. S. Peyrovi H. P. Akbari Kamrani A. A. M. Spahbodi F. M. (2017). Uncertainty, the overbearing lived experience of the elderly people undergoing hemodialysis: A qualitative study. International Journal of Community Based Nursing and Midwifery, 5(1), 13–21. https://www.ncbi.nlm.nih.gov/pmc/articles/PMC5219560/28097174 PMC5219560

[R35] Schouten R. W. Haverkamp G. L. Loosman W. L. Chandie Shaw P. K. van Ittersum F. J. Smets Y. F. C. Vleming L. J. Dekker F. W. Honig A. Siegert C. E. H. (2019). Anxiety symptoms, mortality, and hospitalization in patients receiving maintenance dialysis: A cohort study. American Journal of Kidney Diseases, 74(2), 158–166. 10.1053/j.ajkd.2019.02.017 31027882

[R36] Shi Y. Yang Y. Wang L. Zhang J. (2023). The lived experiences of loneliness of older adults with chronic conditions aging at home: A qualitative systematic review and meta-aggregation. Geriatric Nursing, 51, 274–285. 10.1016/j.gerinurse.2023.03.025 37031579

[R37] Taiwan Society of Nephrology. (2024). 2023 kidney disease annual report in Taiwan. Taiwan Society of Nephrology.https://www.tsn.org.tw/en/twrds_detail.html?year=2023 (Original work published in Chinese)

[R38] Tengku Mohd T. A. M. Yunus R. M. Hairi F. Hairi N. N. Choo W. Y. (2019). Social support and depression among community dwelling older adults in Asia: A systematic review. BMJ Open, 9(7), Article e026667. 10.1136/bmjopen-2018-026667 PMC6661578 31320348

[R39] United States Renal Data System. (2025). End stage renal disease: International comparisons: Highlights. In *2024 USRDS annual data report* (Chap. 11). https://usrds-adr.niddk.nih.gov/2024/end-stage-renal-disease/11-international-comparisons

[R40] Zheng S. Masuda T. Matsunaga M. Noguchi Y. Ohtsubo Y. Yamasue H. Ishii K. (2022). Cultural differences in social support seeking: The mediating role of empathic concern. PLOS One, 16(12), Article e0262001. 10.1371/journal.pone.0262001 PMC8718000 34969056

[R41] Zhou W. Li Y. Ning Y. Gong S. Song N. Zhu B. Wang J. Zhao S. Shi Y. Ding X. (2021). Social isolation is associated with rapid kidney function decline and the development of chronic kidney disease in middle-aged and elderly adults: Findings from the China health and retirement longitudinal study (CHARLS). Frontiers in Medicine, 8, Article 782624. 10.3389/fmed.2021.782624 34926526 PMC8674531

